# Time trend of Brazilian hospital admissions and deaths due to asthma among children and teenagers, 1998–2019

**DOI:** 10.1371/journal.pone.0248472

**Published:** 2021-03-15

**Authors:** Luiza Gabriela de Araújo Fonseca, Rêncio Bento Florêncio, Illia Nadinne Dantas Florentino Lima, Lucien Peroni Gualdi

**Affiliations:** Programa de Pós Graduação em Ciências da Reabilitação, Faculdade de Ciências da Saúde do Trairi (FACISA)/Universidade Federal do Rio Grande do Norte (UFRN), Santa Cruz, Rio Grande do Norte, Brazil; Srebrnjak Children’s Hospital, CROATIA

## Abstract

**Background:**

Asthma is one of the most prevalent non-communicable diseases worldwide. The aim of this study was to characterize the distribution of Brazilian hospital admissions due to asthma among children and teenagers between 1998 and 2019, as well as to analyze hospital admission incidence and mortality rate during the period according to the geographic region, age group and gender.

**Methods:**

This is a descriptive time trend study using secondary data regarding hospital admissions and lethality registered in the Brazilian System of Hospital Information of the Brazilian Public Health System (*SIH*/*SUS*) due to asthma (ICD-10) in subjects aged from 0 to 19 years old between 1998 and 2019. The following variables were collected: number and place of hospital admissions classified by the ICD-10, absolute values and frequency by age group, gender and lethality. Statistical analysis was performed by GraphPad Prism version 5.0 software.

**Results:**

The total number of hospital admissions due to asthma was 3,138,064. It was observed that children aged between 1 to 4 years, living in the Northeast region and males showed the highest number of hospitalizations. A 74.37% reduction over a 21-year period was found. The lethality rate found in the study was 0.06, with the highest rates being from the Northeast region, males and < 1-year-old.

**Conclusion:**

Hospital admissions were more prevalent in young children, male gender and in the Northeast region. A decrease of hospital admissions and lethality rate was observed in all groups over time. This profile is important for implementing government strategies to lower hospital admissions and decrease costs.

## Background

Asthma is one of the most prevalent non-communicable diseases worldwide [1]. It is estimated that 300 million people of all ages are diagnosed with asthma around the world [2]. However, there is a wide variation in prevalence, severity and mortality according to geographic location [3]. Although the highest prevalence of asthma (> 20%) is found in developed countries, studies have shown that the prevalence of childhood asthma in Latin America varies between 4% and 30%, and is above 10% in almost all countries [4]. Cardoso and colleagues evaluated the number of hospitalizations due to asthma as well as its costs in a period between 2008 and 2013 in Brazil, finding that more than one million hospital admissions occurred due to asthma in the period with an average cost of USD $160 per hospitalization [4].

Studies have also shown that the incidence and prevalence of asthma between genders differs over the lifetime. Prepubertal males present higher incidence, prevalence and hospitalization rates when compared to females of the same age; however, this pattern reverses during adolescence and remains until the 5^th^ decade of life [5]. Although Brazil has one of the highest prevalence of asthma in the world, studies reporting the number of hospital admissions and mortality rates according to geographic region, age group and gender are still scarce. Thus, it is noteworthy to highlight the necessity to identify the most vulnerable population to prevent avoidable hospitalizations and to investigate the distribution of Brazilian hospital admissions according to asthma profile in aiming to elaborate new preventive strategies to minimize hospital costs. Furthermore, Brazil has shown advances in access to asthma treatment since the first decade of this century due to the implementation of a national public healthcare policy by the Ministry of Health across all of Brazil [6].

The information technology (IT) department of the Unified Health System (*DATASUS*) provides a free and reliable platform through the hospital information system of the Brazilian Public Health System (*SIH*/*SUS*) for processing hospital admission authorization information such as number and costs and mortality rate [7] according to geolocation [8], age and gender. In view of the above, this study aims to characterize the distribution of Brazilian hospital admissions classified by ICD-10 in children between 1998 and 2019, as well as to analyze hospital admission incidence and mortality rate due to asthma in the last 21 years according to the geographic region, age group and gender in Brazil.

## Methods

### Study design

This is a descriptive time trend study using secondary data regarding the hospital admission and lethality rate registered in the Brazilian Hospital Information system of the Brazilian Public Health System (*SIH*/*SUS*) due to asthma classified by the ICD-10 including subjects from 0 to 19 years old. All admissions from private or public services linked to the *SUS* from 1998 and 2019 were assessed.

### Data extraction

Data were extracted from the *SIH*/*SUS* provided by the Health Surveillance Bureau of the Brazilian Ministry of Health through its open access webpage available at the Department of Informatics of the Unified Health System (*DATASUS*). The following variables were collected: number of hospital admissions and the lethality rate classified by the ICD-10 in a period between 1998 and 2019.

**Fig 1:**
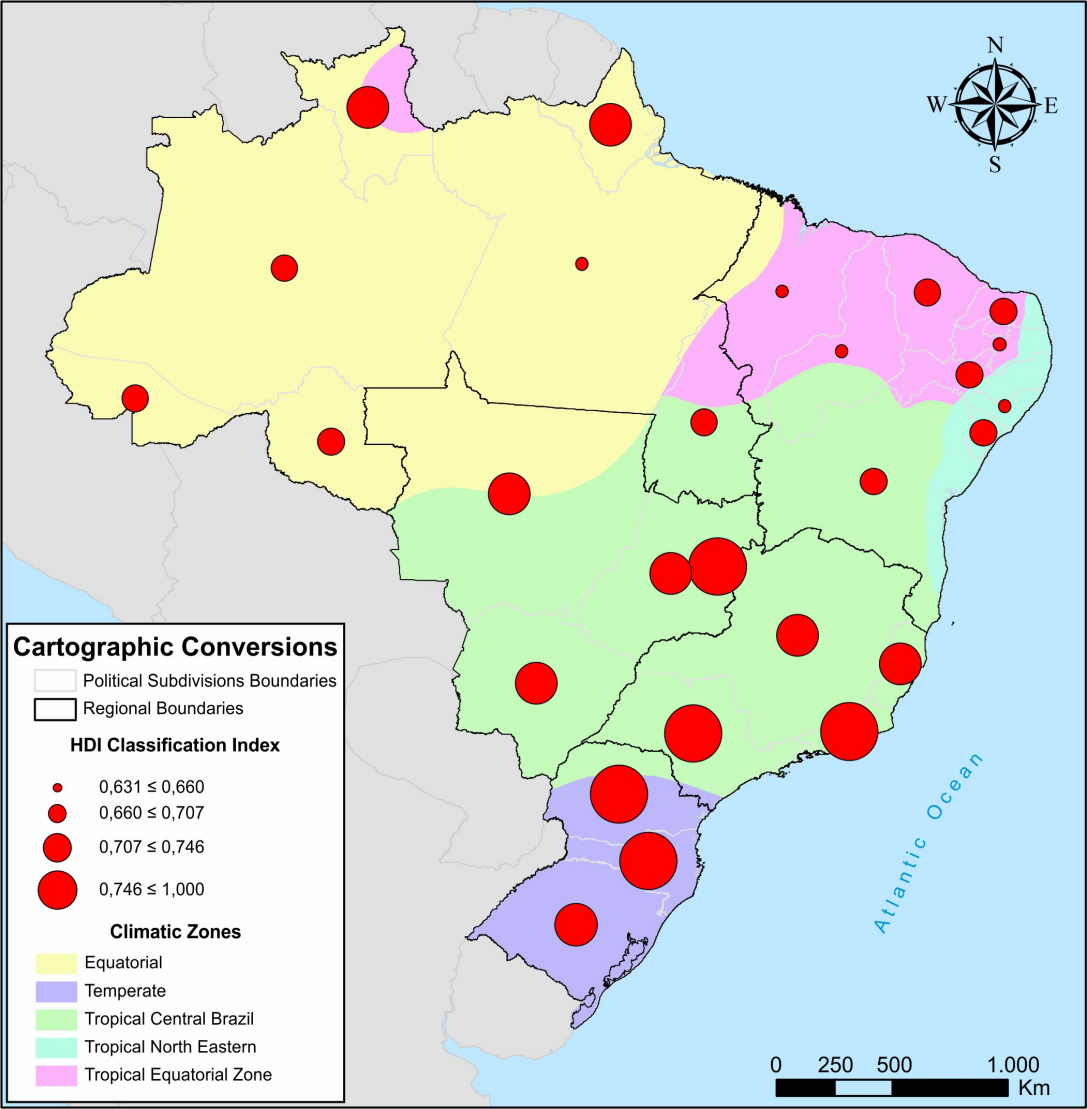
Brazilian geographic map according to resident population, climate and human development index. Source: Brazilian Institute of Geography and Statistics (*IBGE*).

Absolute values and the frequency of hospital admissions and the number of deaths recorded between 1998 and 2019 of the data set were grouped according to gender, age and region. The total population according to region and age group is shown in Table 1, and the mean human development index (HDI) and weather characteristics for each Brazilian region are shown in Fig 1 (ArcGIS software, version 10.5). The data from 1998 to 2003, 2004 to 2009, 2010 to 2015 and 2016 to 2019 were grouped in the longitudinal analyzes.

**Table 1:** Hospital admissions according to age and living region between 1998 and 2019.

Age range		Brazil	Brazilian Region				
	Resident population	Hospitalization (%)	North^(^*^)^	Northeast^(^^*β*^^)^	Southeast ^(^^#^^)^	South ^(^^&^^)^	Midwest ^(^^+^^)^
<1 year	2,879,916	463,906 (16,11)	37,650 (1.2)^***β***^^#^^&^	158,359 (5.05)^+^^*^^&^	158,537 (5.05)^&^^*^^+^	75,042 (2.39)^*^^β^^#^^+^	34,039 (1.08)^***β***^^&^^#^
**1 to 4**	**11,164,677**	**1,531,463 (13,72)**	***141*,*399 (4*.*51)***^***β***^^#^	*643*,*412 (20*.5)^*^^#^^&^^+^	*428*,*716 (13*.66)^*^^β^^&^^+^	***222*,*507 (7*.*09)***^***β***^^#^^+^	***94*,*234 (3*.*0)***^***β***^^&^^#^
5 to 9	15,233,147	671,495 (4,41)	58,352 (1.86)^***β***^^#^^&^	287,638 (9.17)^*^^#^^&^^+^	179,321 (5.71)^*^^β^^&^	95,979 (3.06)^*^^β^^#^^+^	49,737 (1.58)^***β***^^&^^#^
10 to 14	17,463,169	284,785 (1,63)	26,657 (0.85)^***β***^^#^	140,949 (4.49)^*^^#^^&^^+^	55,370 (1.76)^*^^β^^+^	39,448 (1.26)^***β***^	22,150 (0.71)^***β***^^#^
15 to 19	17,282,045	186,415 (1,08)	21,556 (0.69)^***β***^	89,671 (2.86)^*^^#^^&^^+^	26,799 (0.85)^***β***^	30,595 (0.97)^***β***^^+^	17,631 (0.56)^***β***^^&^
**Total**	**64,022,954**	**3,138,064 (4,90)**	**285,614 (9.10)**	**1,320,029 (42.07)**	**848,743 (27.05)**	**463,571 (14.77)**	**217,791 (6.94)**
P value			<0.001	<0.001	<0.001	<0.001	<0.001
**Resident population**	64,022,954		6,799,560	19,542,303	24,411,806	8,438,583	4,830,702
**Hospitalization (%)**	3,138,064 (4,90)		285,614 (2,08)	1,320,029 (3,29)	848,743 (1,76)	463,571 (2,64)	217,791 (1,95)

Datashown as absolute values and frequency (%)

*North

^**β**^Northeast

^#^Southeast

^&^South

^+^Midwest regions.

Comparisons among age groups and living region were made by Two-way ANOVA and Tukey post hoc test. Ignored data is not shown in the table. Source: Brazilian Institute of Geography and Statistics (IBGE) and Ministry of Health, Brazilian Public Health System—Hospital Information System (SIH / SUS).

All data are provided by the hospital information system (*SIH*/*SUS*) in an online platform (link: http://datasus.saude.gov.br/), and were collected from March to April, 2020. Regions and age group analysis by resident population followed the standards of the general analysis, of the Brazilian Institute of Geography and Statistics (IBGE) platform (link: http://ibge.gov.br), according to the demographic census of 2010.

### Data analysis

Extracted data were stored in a Microsoft Excel 2013 program spreadsheet. Statistical analysis was performed by the GraphPad Prism version 5.0 software program. Data normality was assessed by the Kolmogorov-Smirnov test. Comparisons among the groups were performed by the Kruskal-Wallis test, Dunn’s Multiple Comparison post hoc test and the Mann-Whitney test. Intra and intergroup analysis was performed by Two-way ANOVA with a Tukey post hoc test. A p-value < 0.05 was considered significant.

### Ethical aspects

All data from the study are public with free access and may be accessed at *DATASUS* (http://datasus.saude.gov.br/) and *IBGE* (http://ibge.gov.br). Ethical approval was not required in accordance with the Brazilian National Health Council (Resolution No.510 from April 07^th^, 2016), which regulates the National Research Ethics Committee (*CONEP*). Patients’ confidentiality was preserved in accordance with *CONEP*.

## Results

The total number of hospital admissions due to asthma was 3,138,064 between 1998 and 2019 in Brazil. When we observed the temporal trend we found a significant decrease in absolute values when we compared 1998 with 2019 (216,477 vs. 55,489, respectively, p = 0.001), constituting a 74.37% reduction over a 21-year period.

### Hospital admissions according to age and living region

When comparing total hospital admissions according to age, it was observed that children between 1 and 4 years showed the highest number of hospitalizations due to asthma (1,531,463; 48.80%), followed by children between 5 and 9 years (671,495; 21.40%); and younger than 1 year (463,906; 14.78%) (Table 1). A decrease in hospital admissions according to age was also observed when we compared 1998 to 2019 varying from 85.56% (15,494 vs. 2,237; p<0.0001) in the group aged between 15 and 19 years, and 61.73% (42,267 vs. 16,176; p = 0.009) in the group aged between 5 and 9 years.

When we assessed hospital admissions grouped according to living region it was found that the Northeast region showed the highest incidence for hospital admissions due to asthma (1,320,029; 42.07%), followed by the Southeast (848,743; 27.05%) and South regions (463,571; 14.77%). All significant differences among regions are shown in Table 1.

### Hospital admissions according to sex

**Fig 2:**
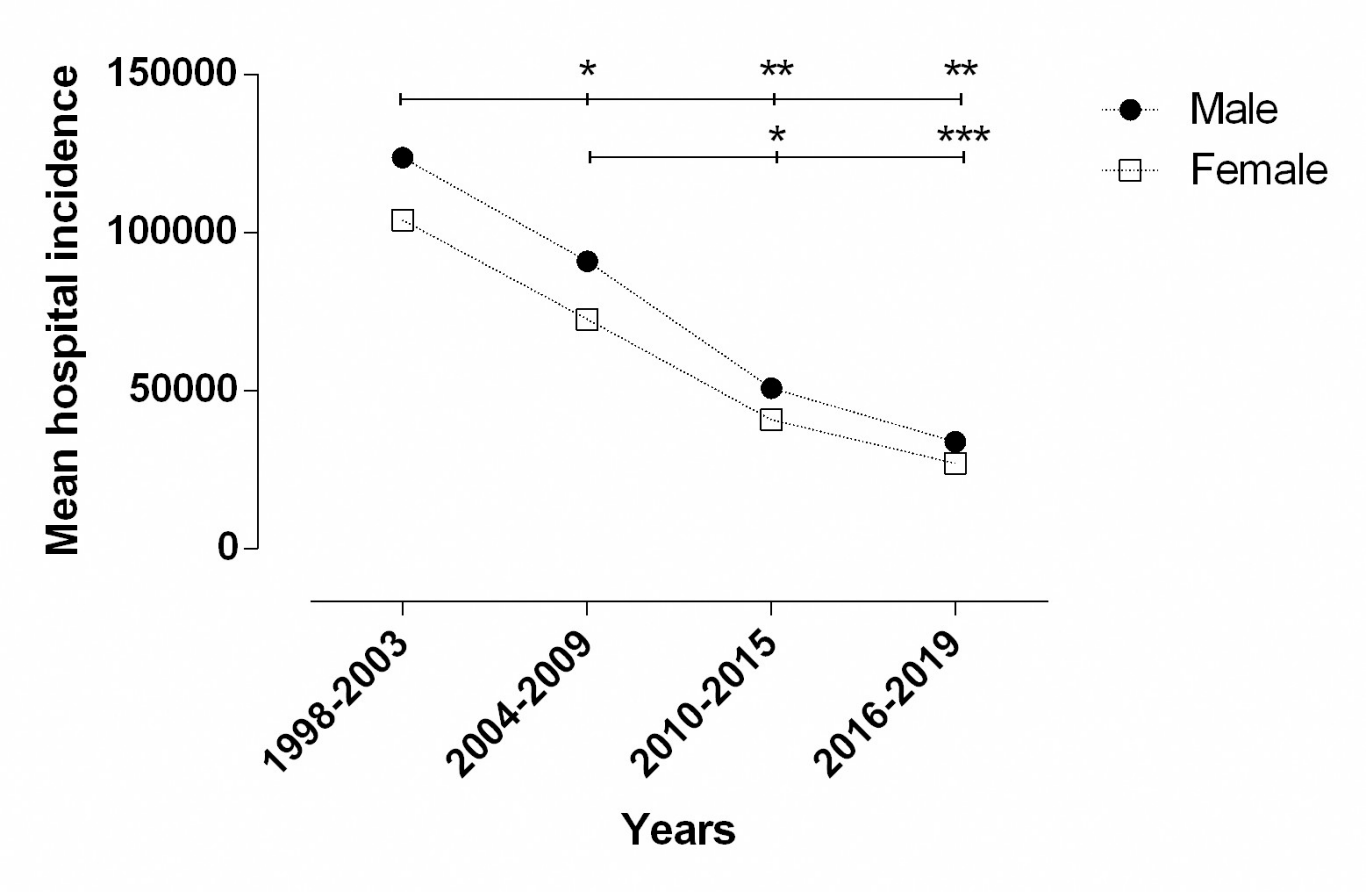
Mean hospital admission incidence due to asthma according to gender between 1998 and 2019 in Brazil. Statistical difference calculated by Two-way ANOVA with Tukey post hoc test; *statistically significant difference between period: *p = 0.01, **p = 0.001, ***p = 0.004. Closed symbol: males; open symbol: females. Source: Ministry of Health, Brazilian Public Health System—Hospital Information System (*SIH*/*SUS*).

Although males showed higher total incidence of hospital admission when compared to females in different Brazilian regions (1,727,198; 55.04% vs. 1,410,858; 44.96%, respectively; p = 0.167), there was no significant difference between genders. When we compared 1998 to 2019 we found a significant decrease for both males and females (115,675 vs. 30,842, p = 0.001; 100,800 vs. 24,647, p = 0.0007; respectively). Fig 2 shows the significant differences between genders according to the time periods.

**Fig 3:**
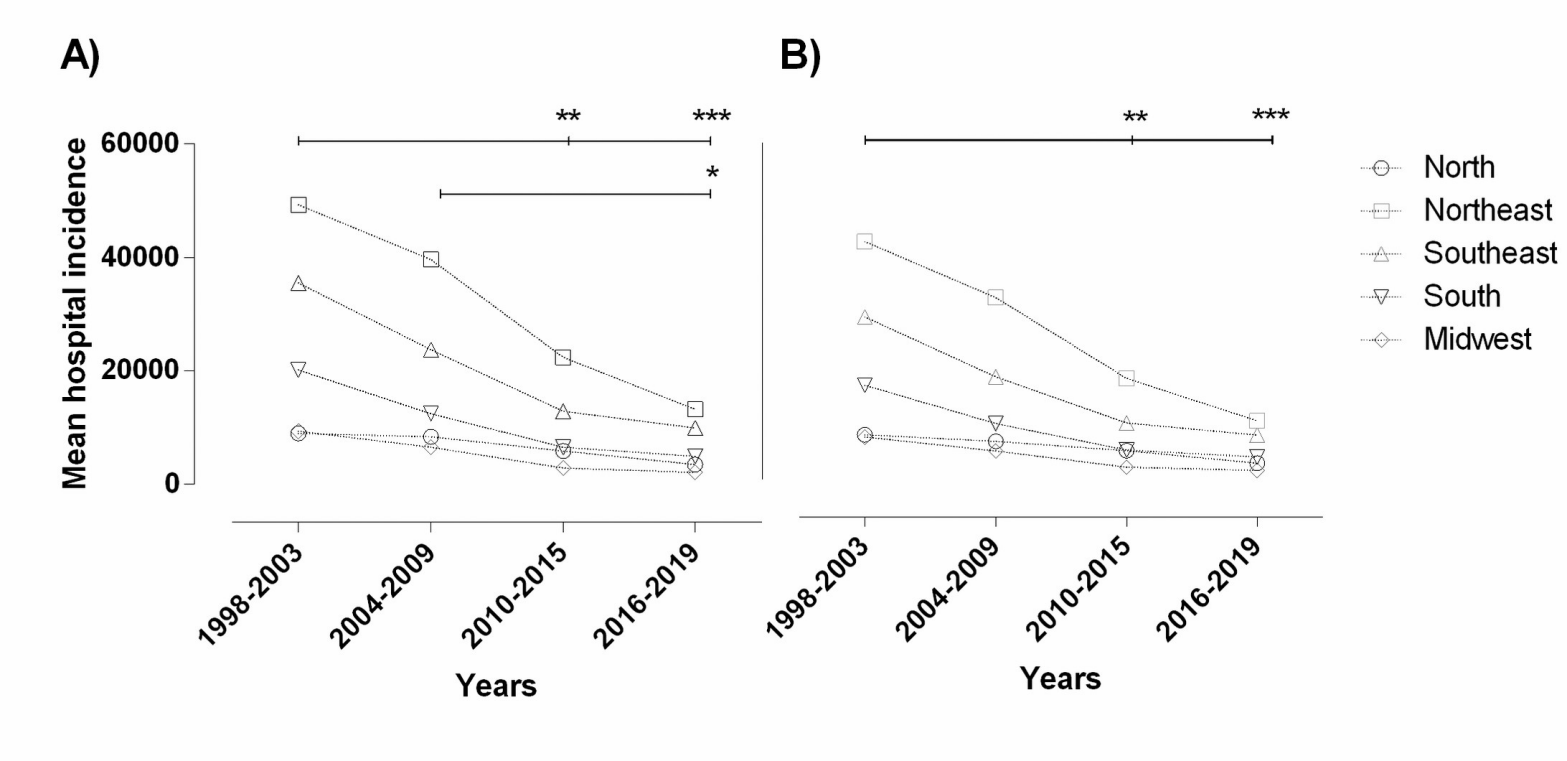
Mean incidence of asthma hospitalizations according to living region and gender between 1998 and 2019 in Brazil. Statistical difference calculated by Two-way ANOVA with Tukey post hoc test; *statistically significant difference between period: *p = 0.044, **p = 0.010, ***p = 0.002. A) males and B) females. Source: Ministry of Health, Brazilian Public Health System—Hospital Information System (*SIH*/*SUS*).

In the longitudinal analysis, we found that the Northeast region showed higher hospital admissions in all study periods for both genders, while the Midwest region showed the smallest numbers of hospital admissions for both genders. Fig 3A and 3B show the significant differences among the study periods for males and females, respectively.

### Lethality according to living region and age group

The number of registered deaths caused by asthma between 1998 and 2019 was 1,977 (1,022 in males and 955 in females). Mean national lethality during the study period was 0.06. There was no significant difference in the lethality mean between 1998 and 2019 (p = 0.224).

**Fig 4:**
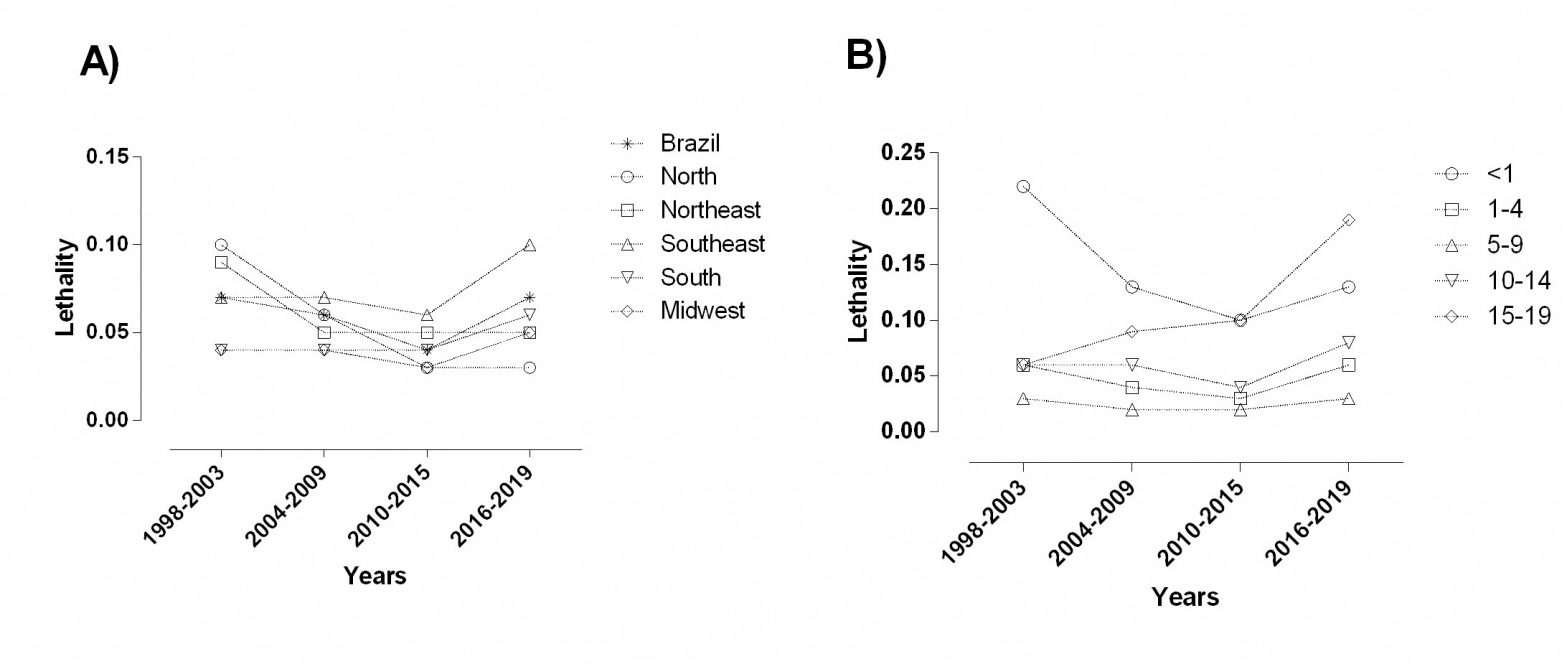
Lethality due to asthma according to A) living region and, B) age group during the study period. Source: Ministry of Health, Brazilian Public Health System—Hospital Information System (*SIH*/*SUS*).

When we assessed the lethality rates according to region between 1998 and 2019, we found that the Southeast region showed the highest mean lethality (0.073), followed by the Northeast (0.062) and the Midwest region (0.039). There was no significant difference when we compared the mean lethality according to living region among the study periods (Fig 4A).

We also considered lethality rates according to age group between 1998 and 2019. The higher mean lethality was found in the <1 year group (0.147), followed by the 15 to 19-year-old group (0.101). There was a significant difference between the <1-year-old group and the 1–4 year-old (p = 0.012), the 5–9 year-old (p = 0.002) and 10–14 year-old (p = 0.03) groups. Although there was no significant difference between the study periods, the <1 year-old group showed a higher lethality reduction, while those in the 15–19 year-old group showed an increase in the lethality rate over the years (4B).

**Fig 5:**
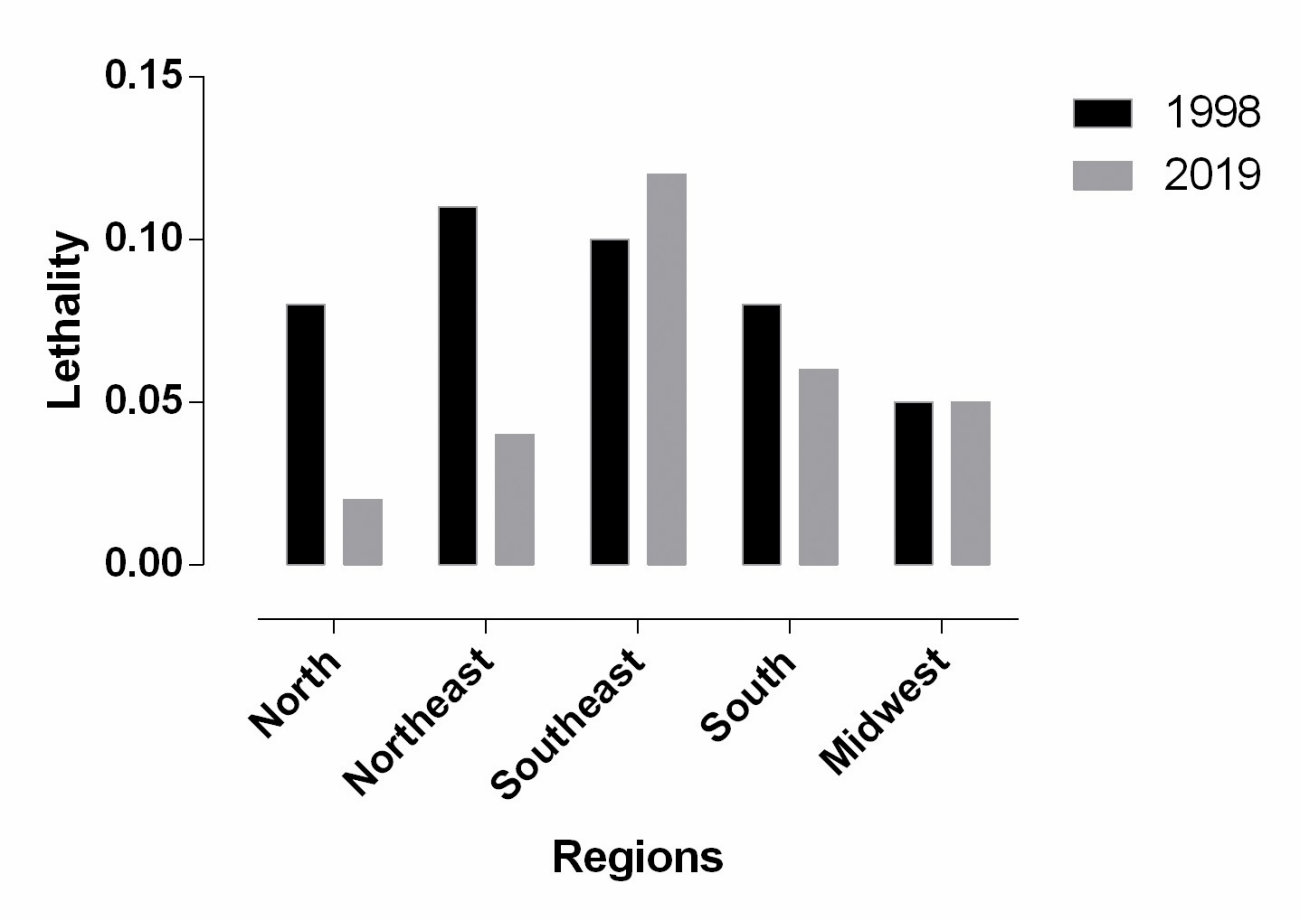
Lethality due to asthma between 1998 and 2019. Source: Ministry of Health, Brazilian Public Health System—Hospital Information System (*SIH*/*SUS*).

Although we did not find a significant difference in the comparison between 1998 and 2019, the Northeast region showed the highest lethality rate reduction (0.11 vs. 0.04; p = 0.890), while the Southeast region showed an increase (0.10 vs. 0.12; p = 0.999) (Fig 5). Lethality was determined by the ratio of the number of deaths to the number of hospital admissions approved and authorized (which were computed as hospital admissions) between 1998 and 2019 and multiplied by 100.

### Lethality according to gender

**Fig 6:**
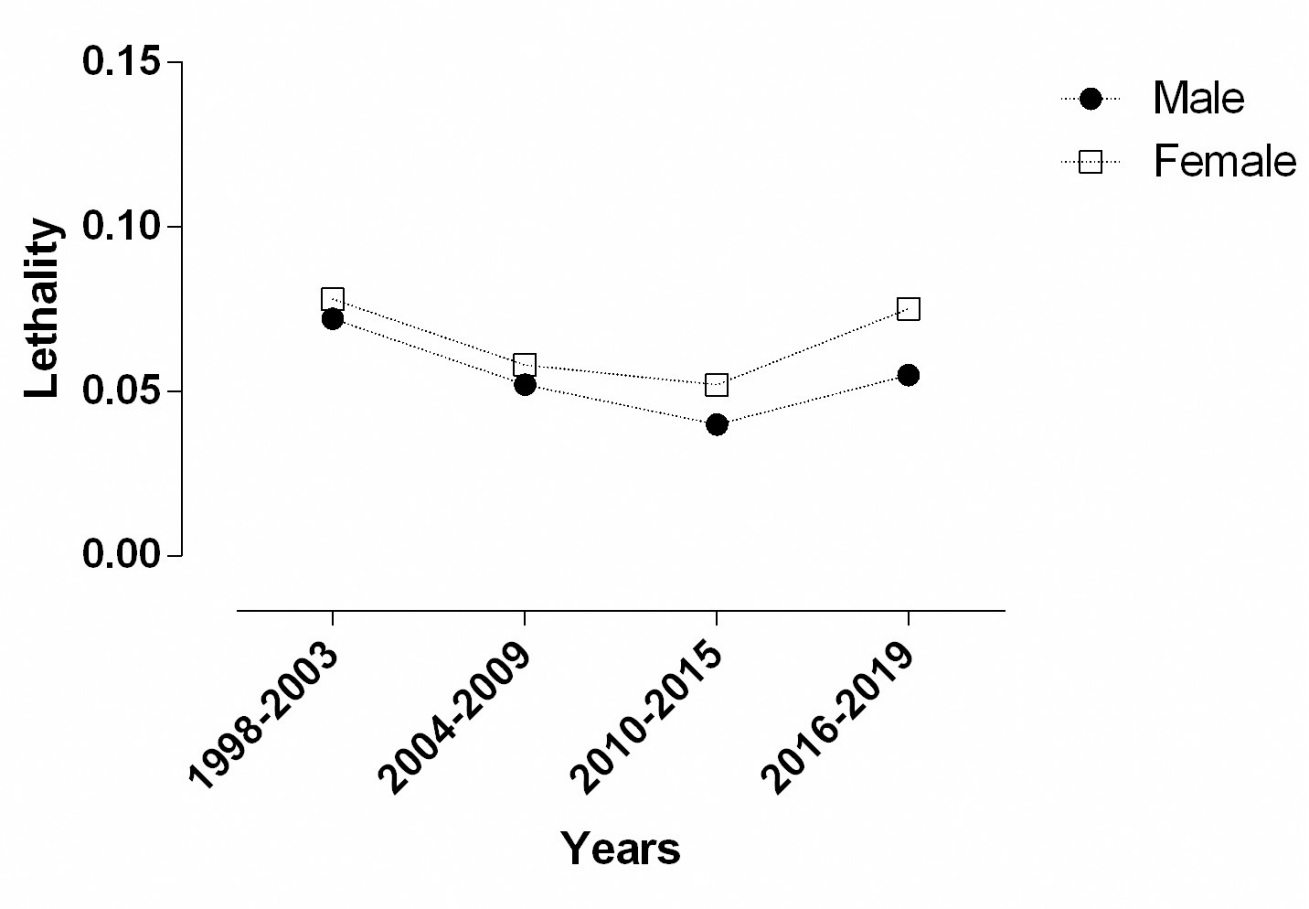
Lethality due to asthma according to gender between 1998 and 2019 in Brazil. Closed symbol: males; open symbol: females. Source: Ministry of Health, Brazilian Public Health System—Hospital Information System (*SIH*/*SUS*).

Similar absolute numbers were found in the study period when death numbers between genders were considered (51.69%; 1,022 vs. 48.31%; 955 in males and females, respectively). There was no significant difference in the mean lethality when we compared females and males (0.07 vs. 0.05, p = 0.226; respectively). Fig 6 shows lethality according to the study period in males and females.

## Discussion

In this study we found 3,138,064 hospital admissions due to asthma in the studied population; however, there was a reduction of 74.37% in hospitalizations between 1998 and 2019. The highest number of hospital admissions occurred in children between 1 and 4 years old and those living in the Northeast region. General lethality was also higher in those individuals from the Northeast region. We also found greater numbers of hospital admissions in the male gender; however, lethality was higher in the female gender.

To our knowledge, this is the first study which has analyzed hospital admissions in different age groups of children and adolescents, as well as considering the living region and separated by gender. The authors of a study performed in four Brazilian state capitals observed no significant difference in hospital admissions due to asthma in three different age groups (12–17 years, 18–40 years and ≥ 41 years) in period of one year [9]. A multicenter prospective observational study conducted in the USA [10] found a higher prevalence of hospitalizations due to asthma in children (6–12 years; 9–22%) when compared to adolescents (13–17 years; 10–17%) and adults (≥ 18 years; 5–15%); this finding is consistent with our results, although the age groups are not completely similar.

When we assessed hospital admissions by region we found that the Northeast region showed the highest number of hospitalizations due to asthma, even greater than the national average [4]. These findings may be explained by the socioeconomic characteristics of the Northeast region which is considered the least developed and poorest region of the country [11]. This is further due to the high treatment costs as severe asthma consumes almost 25% of the family income in those least favored patients, although WHO’s recommendation does not exceed 5% of family income [12]. Moreover, Mallot et al. suggested a relationship between the tropical weather and the high prevalence of asthma [13].

The present study generally observed a reduction around 74% in the number of hospitalizations due to asthma in Brazil in the last 21 years. Such a finding corroborates with the Ministry of Health [14] which showed 62% less hospitalizations due to asthma between 2000 and 2011, and by Cardoso et al. (2017)^4^ who found a reduction of 36% in absolute numbers when analyzing the period from 2008 to 2013.

We found a lethality of 0.06 in the study sample. Moreover, we also found a decrease of 81.5% in the comparison between 1998 and 2009. Pitchon et al. (2019) [15] also found a reduction in asthma mortality, but the reasons for the temporal trend were not investigated. We believe that the implementation of the National Asthma Control Plan [6] to finance drugs for asthma control for severe, moderate and mild asthmatic subjects implemented in 2000 may have influenced disease control and the hospital admission decrease during the study period. Such a decrease was also observed in the study performed by Sole et al. (2015) [16].

The authors believe that the implementation of public health strategies such as family health, humanization and community agents (which provides better monitoring of asthmatic patients) are partially responsible for the reduced hospitalization and lethality in these patients [17]. For example, the implementation of a program for adequate treatment and distribution of medicines in Salvador city located in the Northeastern region resulted in improved control, quality of life and family income [18]. However, better control of such strategies is still needed across all of the Brazilian territory.

A total of 37% of the total deaths occurred in those aged 1–4 years, which corroborates the study performed by Pitchon et al. (2019) in which the same pattern was observed with 2,255 cases (45%) in children of a similar age [15]. Other studies in Brazil have also reported an asthma mortality decrease in pediatric patients (≤ 19 years of age), as was subsequently reported for the city of Rio de Janeiro [19] and in general since the 1990s [20]. Other studies have also shown that the inpatient mortality rate was higher in the Southeastern and South regions.

In the present study we found that the Northeast region presents higher hospitalization numbers and mortality rate, followed by the Southeast and South regions. It is important to note that such differences may be explained by the study period, as Cardoso et al. [4] calculated mortality rate based on the 2010 findings, while our analysis was performed between 1998 and 2019. Several authors have speculated that a possible explanation may be related to the reduction of infant mortality in all Brazilian regions due to fecundity decrease, as well as public strategies, sanitation and parental education improvement, among other aspects mainly in the poorest Brazilian regions like the Northeast and North [15].

We generally observed a predominance of deaths in female children when compared to males in all age groups. Pitchon et al. (2019) [15] found a predominance of deaths in females only when analyzing the subjects between 10 and 19 years old. These differences are still not well understood in the literature [21], but few studies indicate that boys are more likely to develop the disease in early childhood, while girls are more likely at older ages, mainly due to testosterone [22, 23].

The main limitation in our study is the descriptive time trend design in which we analyzed secondary data, which may include wrong or underreported diagnoses. However, the *DATASUS* system stores official information from the Brazilian government and may be considered as an asthma overview in the country. Due to the fact that the study population includes infants, another limitation is the non-differentiation of wheezing infants, mainly in those children under two years old [24]. However, we included all data from asthmatic children from the Brazilian government’s official platform.

## Conclusion

Despite the high number of reported cases of hospitalizations and deaths due to asthma throughout Brazil, it was possible to observe that the country presented an important reduction in such impairments. Children between 1 and 4 years old living in the Northeast region showed the highest numbers of hospital admissions and deaths in comparison to other Brazilian regions. Thus, public health policies must consider these groups in the development of new asthma prevention strategies.
